# Pyruvate formate lyase regulates fermentation metabolism and virulence of *Streptococcus suis*

**DOI:** 10.1080/21505594.2025.2467156

**Published:** 2025-02-20

**Authors:** Qingying Fan, Haikun Wang, Shuo Yuan, Yingying Quan, Rishun Li, Li Yi, Aiqing Jia, Yuxin Wang, Yang Wang

**Affiliations:** a College of Animal Science and Technology, Henan University of Science and Technology, Luoyang, Henan, China; b College of Life Sciences, Northwest A&F University, Yangling, Shaanxi, China; c Henan Provincial Engineering Research Center for Detection and Prevention and Control of Emerging Infectious Diseases in Livestock and Poultry, Luoyang, Henan, China; d College of Life Science, Luoyang Normal University, Luoyang, China; e Guangdong Haid Institute of Animal Husbandry and Veterinary, Guangzhou, P.R. China

**Keywords:** *Streptococcus suis*, pyruvate formate lyase, bacterial metabolism, Virulence

## Abstract

*Streptococcus suis*, a zoonotic pathogen, is commonly found as a commensal bacterium in the respiratory tracts of pigs. Under specific conditions, it becomes invasive and enters the blood, causing severe systemic infections. For *S. suis*, effective acquisition of carbon sources in different host niches is necessary for its survival. However, as of now, our understanding of the metabolism of *S. suis* within the host is highly restricted. Pyruvate formate lyase (PFL) plays a crucial role in bacterial survival of in glucose-limited and hypoxic host tissues. Here, we investigated the physiological and metabolic functions of PFL PflB in *S. suis* and elucidated its pivotal role in regulating virulence within the mucosal and blood niches. We demonstrate that PflB is a key enzyme for *S. suis* to support mixed-acid fermentation under glucose-limited and hypoxic conditions. Additionally, PflB is involved in regulating *S. suis* morphology and stress tolerance, and its regulation of capsular polysaccharide content depends on dynamic carbon availability. We also found that PflB is associated with the capacity of *S. suis* to cause bacteremia and persist in the upper respiratory tract to induce persistent infection. Our results provide highly persuasive evidence for the relationship between metabolic regulation and the virulence of *S. suis*.

## Introduction

*Streptococcus suis*, a zoonotic pathogen, is a facultative anaerobe that can colonize the upper respiratory tract of pigs, including the tonsils and nasal cavity, without causing any symptoms [1]. When *S. suis* breaches the mucosal barriers, such as the tracheal epithelial barrier or blood–brain barrier, and gains access to the bloodstream, it becomes an invasive pathogen, causing bacteremia, endocarditis, arthritis, pneumonia, and even streptococcal toxic shock syndrome [2]. Regardless of whether it establishes asymptomatic colonization or causes invasive infection, *S. suis* must undergo adaptive changes to thrive within different host niches [1,3]. Accumulating evidence suggests a close correlation between carbohydrate availability in different ecological niches and the regulation of bacterial virulence [4–6]. Ferrando et al. preliminarily indicated that carbohydrate availability can affect the regulation of *S. suis* virulence, thereby influencing the switch from asymptomatic to pathogenic association [7]. The porcine upper respiratory tract niche, such as the tonsil, provides a continuous environment for *S. suis* colonization and is considered a reservoir for *S. suis* replication [1]. However, free glucose, which is preferred by bacteria for metabolism, is limited to this specific niche [8,9]. Instead, the galactose in the O/N-linked glycans derived from host glycoproteins is the primary carbon source for bacterial metabolism in this niche [10]. To establish an invasive infection in vivo, *S. suis* must traverse the mucosal barrier of the host and gain access to the bloodstream. Once within the bloodstream, it can proliferate and disseminate along with the circulating blood, unlike in tissues where ample glucose is readily available [11,12].

**Figure 1.**
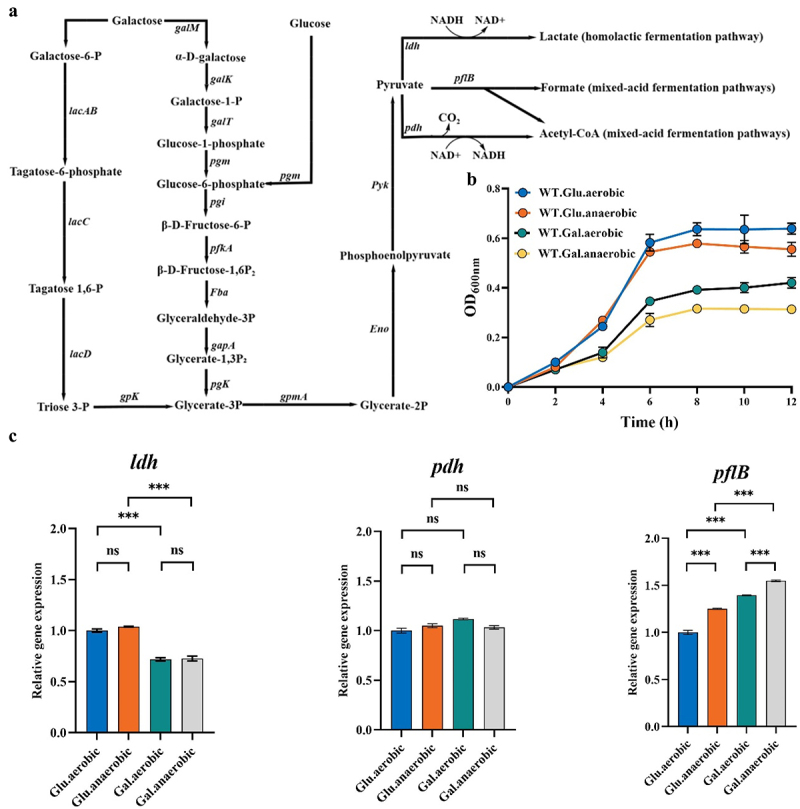
PFL supports the survival of *S. suis* under glucose-limited and conditions. (a) The metabolic pathways of glucose and galactose in *S. suis*. (b) Growth performance of *S. suis* under different culture conditions. (c) Transcription of *ldh*, *pdh*, and *pflB* in *S. suis* under different culture conditions. (ns: no significance; *, *p* < 0.05; **, *p* < 0.01; ***, *p* < 0.001).

In bacteria, carbohydrates are primarily metabolized through glycolysis and the pentose phosphate pathway (PPP) [13]. Pyruvate is a key branch point in glycolysis. Depending on the fate of the pyruvate, it can be reduced to lactate and CO_2_ by lactate dehydrogenase (LDH), resulting in homolactic fermentation [12]. Alternatively, mixed acid fermentation can occur via the action of the pyruvate dehydrogenase complex (PDHc) and pyruvate formate lyase (PFL) [14]. Under aerobic conditions, PDHc converts pyruvate to acetyl-CoA and CO_2_ while reducing NAD^+^ to NADH by providing H^+^. Under microaerobic and anaerobic conditions, PFL converts pyruvate to acetyl-CoA and formate [15,16]. The availability of oxygen and the type and content of free sugars in the host determine whether the bacteria will undergo homolactic or mixed acid fermentation (Figure 1a) [17]. Studies on *Streptococcus pneumoniae* metabolism have revealed its ability to adapt to different host niches through the regulation of pyruvate metabolism. PFL plays a crucial role under anaerobic and glucose-limited conditions by controlling bacterial metabolic processes, influencing cell membrane lipid composition, and contributing to the virulence of *S. pneumoniae* in different tissues [17]. To date, the role of PFL in the growth and metabolism of *S. suis* and its potential impact on the virulence, similar to that reported for other bacteria, remains unknown.

In this thesis, we investigated the changes in the growth and metabolites of *S. suis* under different carbon sources and ventilation conditions in vitro. Our findings demonstrate that PFL plays a crucial role in supporting the survival of *S. suis* under glucose-limited and hypoxic conditions. Additionally, we generated a PFL PflB deletion mutant of *S. suis* to assess the impact of PflB on the growth, metabolism, morphology, and virulence of *S. suis* at the cellular and host levels of *S. suis* infection. These results further emphasize the subtle role of metabolic regulation in the virulence of *S. suis*.

## Materials and methods

### Ethics statement

Mice were fed and all experimental procedures were conducted in accordance with the regulations for the Administration of Laboratory Animals approved by The State Council of the People’s Republic of China. The experimental protocol and euthanasia procedure involving experimental animals in this study were approved by the Laboratory Animal Supervision and Management Committee of the Henan University of Science and Technology (approval number: SKKUIACUC-23-08-08-6; SKKUIACUC-24-07-11-4). The study is also in accordance with ARRIVE guidelines.

### Bacterial strains, plasmids, cell lines, and animals

*S. suis* serotype 2 ZY05719, referred to as the WT in this study, was used. *S. suis* was cultured in TSB (BD Biosciences, CA, USA) at 37°C under aeration unless otherwise stated. Under restrictive conditions, it was cultured in a compound medium (TOPBIO, Shandong, China) without exogenous carbon as the basal medium and exogenously supplemented with 1% glucose or 1% galactose (Aladdin, Shanghai, China). Hep-2 cells were purchased from the Cell Bank of the Chinese Academy of Sciences and were cultured in complete medium of DMEM (high glucose) or F12K (1% glucose or galactose). Female BALB/c mice (6 weeks old) were provided by the Experimental Animal Center of Zhengzhou University (Zhengzhou, China). Female piglets (12 weeks old) were sourced from a farm where no pertinent porcine pathogens were detected. The farm owner of Songxian Jinfoping Agricultural Technology Co., Ltd., gave verbal consent for the animals to participate in the study.

### Construction and complementation of pflB mutants

Δ*pflB* was constructed using homologous recombination. The *pflB* gene was cloned into the pSET4s plasmid, which is an *S. suis*–*E. coli* shuttle vector, to generate the knockout vector pSET4S-*pflB*. Subsequently, electroporation was performed. Deletion mutant was constructed by homologous recombination twice, and *pflB* knockout was detected by PCR. The complementary strain CΔ *pflB* was constructed based on the Δ *pflB* strain. The coding sequence of *pflB*, including its upstream promoter, was amplified and subsequently cloned into the pSET2 vector. As mentioned above, this vector (pSET2-*pflB*) was delivered into Δ *pflB* by electroporation. After screening for spectinomycin resistance, *pflB* gene was amplified by PCR to identify CΔ *pflB*.

### Growth curves assay

The overnight culture media of WT, Δ *pflB,* and CΔ *pflB* were transferred to TSB/glucose monosaccharide/galactose monosaccharide medium at a ratio of 1:100 and incubated at 180 rpm and 37°C under aerobic or anaerobic conditions, respectively. For aerobic cultures, bacteria grow in standard culture tubes. For anaerobic ones, the surface of the freshly sterilized culture tube is sealed with 3 ml sterile liquid paraffin to isolate air and create an anaerobic environment. Absorbance at 600 nm was measured every 2 h. Three biological replicates were used for each measurement point, and the growth curves were plotted.

### Quantification of glucose and galactose and fermentation products during growth

The supernatants of WT, Δ *pflB*, and CΔ *pflB* cultured in different monosaccharide media and under different ventilation conditions at different stages (0, 2, 4, 6, 8, and 10 h) were collected. The content of glucose, galactose, and the fermentation products lactate and formate was detected. The contents of glucose and galactose are, respectively, detected by using the Glucose Assay Kit with O-toluidine (Beyotime, Shanghai, China) and the D-Galactose Assay Kit (Jiangsu Aidisheng Biological Technology Co., Jiangsu, China). The metabolites lactate and formate were determined using the Lactic Acid (LA) Content Assay Kit (Solarbio, Beijing, China) and Formate Assay Content Assay Kit (Abcam, USA).

### RNA isolation and real-time quantitative PCR (rt-qPCR)

The WT cultured for 10 h under the conditions of glucose monosaccharide medium in aerobic and anaerobic conditions, and galactose monosaccharide medium in aerobic and anaerobic conditions were collected, respectively. RNA was extracted using an RNA isolator Total RNA Extraction Reagent kit. Reverse transcription was performed using the PrimeScript™RT reagent Kit. RT-qPCR was performed using AceQ Universal SYBR qPCR Master Mix (Q511–02, Vazyme, Nanjing, China). The 16S rRNA amplicon served as the internal control. The transcription levels of key node genes (*ldh*, *pflB*, and *pdh*) in pyruvate metabolism were determined using the relative quantification method (ΔΔCT) as previously reported [18,19]. The primers used are listed in Table 1.

**Table 1.** Primers used in this study.

Primer name	Primer sequence
*16sRNA*-1	GTTGC GAACG GGTGA GTAA
*16sRNA*-2	TCTCA GGTCG GCTAT GTATC G
*ldh*-1	ATCGTTCGTCCAGTCAATATCC
*ldh*-2	GGGTTTGCGAAAGCATCATC
*pflB*-1	CCCTTGACTGGTTGACTGATAC
*pflB*-2	CAGATACCGAAGCCCATGTTAG
*pdh*-1	TTGCGTCGTTATGTAGAG
*pdh*-2	ACAAATGGGTTCCCGTCA
*pflB*-A	CCGCGTCGACCAAATCTTGCCCCGTGTAGACCCCC
*pflB*-B	GAGGGTTCTCCTTTTATGCTTTTGG
*pflB*-C	CCAAAAGCATAAAAGGAGAACCCTCTCAAGCAGACACAA AAGGACTTGCA
*pflB*-D	ACCGGAATTCCGATTTTGAAGATTGCTGTAACGGA
*pflB*-ORF-S	GCTATACTCCAGACCCACTCCTT
*pflB*-ORF-A	TCATCGTAACCGTTGTTCCAG
*pflB*-XY-S	CCAAAACGCCCAAATCTATCT
*pflB*-XY-A	TTCGGCAATTATTGACCCAT
*pflB-F*	CCCAGCTGAGATCTCCTAGGTGTCAACAAAAGTTAAAAC
*pflB-R*	TGCTGCCGGTCACTTAAGAGCTTGACCTGAAACTTC

### The evaluation of the integrity of bacterial cell membrane and cell wall

The culture supernatants of WT, Δ *pflB*, and CΔ *pflB* in the logarithmic growth phase (8 h) were taken. The integrity of cell membranes and cell walls was evaluated using LDH Release Assay Kit (Beyotime, Shanghai, China) and alkaline phosphatase (ALP) test kit (Beyotime, Shanghai, China), respectively.

### Detection of bacterial tolerance to adverse environments

The WT, Δ *pflB*, and CΔ *pflB* in with the same CFU (10^6^ CFU/mL) were used to evaluate their tolerance to adverse environments. For high-temperature stress, 100 μL of each bacterial culture was diluted and plated on TSB agar (TSA), then incubated at 37°C and 40°C for 24 h before counting colonies [20]. For acid stress, bacteria were washed and resuspended in TSB at various pH (7.0, 6.0, 5.0, 4.0), incubated for 45 min at 37°C, plated, and counted after 24 h [21]. For oxidative stress, 20 or 40 mm H_2_O_2_ was added, respectively, to the three kinds of bacterial culture, incubated at 37°C for 20 min, then diluted and plated on TSA for counting [22].

### Scanning electron microscopy (SEM) and transmission electron microscopy (TEM)

For both SEM and TEM, the WT, Δ *pflB*, and CΔ *pflB* in the logarithmic growth phase were collected. For SEM, the bacteria were fixed overnight in 2.5% glutaraldehyde, and then they were washed three times with deionized water and then fixed with 10%, 30%, 50%, 70%, 90%, and 100% absolute ethanol for 10 min each. Finally, the bacteria were dried at room temperature and observed under a scanning electron microscope JSM-7800F (Nippon Electric Co, Tokyo, Japan). For TEM, the bacteria were fixed overnight in a solution containing 2.5% glutaraldehyde. After embedding in epoxy resin and dehydration using propylene oxide, sections were obtained for further processing. These sections were then stained with 1% uranyl acetate and alkaline lead citrate before observation under H-7650 TEM (Hitachi, Tokyo, Japan).

### Quantification of bacteria capsular polysaccharides (CPS)

Quantification analysis of CPS was conducted following the Marie-Rose Van Calsteren’s method [23]. Briefly, overnight cultures of WT, Δ *pflB,* and Δ *pflB* were cultivated separately in media containing glucose and galactose monosaccharides. The bacteria were collected, and the precipitate was washed with PBS before being resuspended in 100 mL of glycine buffer solution (0.1 M pH = 9.2) supplemented with 100 mg of lysozyme for subsequent lysis under constant agitation at 37°C for 8 h. Then, the supernatant was removed by centrifugation at 8,000 × *g* for 20 min. Protein K was added to the supernatant at a concentration of 100 μg/mL and incubated at 55°C for 2 h. A CaCl_2_ solution was then added to the treated solution to achieve a final concentration of 0.1 M and stirred for 1 h. Add one-third volume of absolute ethanol (25%V/V), place at 4°C for 3 h, centrifuge (8,000 × *g*, 20 min) to remove the nucleic acid from the supernatant, and, finally, add three times the volume of absolute ethanol (concentration to 80% V/V) and place at 4°C for more than 16 h after full agitation. The reaction was concluded by subjecting the mixture to centrifugation (8,000 × *g*, 20 min) to facilitate the precipitation of the CPS from the solution.

### Adhesion and invasion assays

Both traditional and modified adhesion/invasion assays were performed [24,25]. The revised version of the adhesion/invasion assay was performed according to Haley Echlin’s methodology [25]. Briefly, Hep-2 cells in high glucose DMEM and three kinds of bacteria (WT, Δ *pflB*, and CΔ *pflB*) in TSB were used for the traditional adhesion/invasion experiments, while Hep-2 cells in F12K (1% glucose or galactose) and three kinds of bacteria with galactose or glucose as the carbon source were used for the modified experiments. For each type adhesion/invasion experiment, the CFUs of WT, Δ *pflB*, and CΔ *pflB* were adjusted to be uniform, the MOI (bacteria: cells) ratio was 100:1. After the 3-h infection process was completed, PBS was used to remove non-invasive and non-adherent bacteria. For invasion assay, 100 U/mL penicillin and 0.1 mg/mL streptomycin were added to the wells and co-incubated for 2 h in 5% CO_2_ incubator to remove the bacteria adhered to the cell. The cells were then washed twice with PBS, lysed with chilled ultrapure water, plated and counted for invasive bacteria. For adhesion assay, antibiotic treatment was not required. The cells were directly lysed with chilled ultrapure water and plated. The number of bacterial clones was subtracted from the previous invasive count to obtain adhered bacteria. The adhesion and invasion assays were repeated three times, with each experiment done in triplicate.

### Whole blood killing assay

The porcine blood utilized in this assay was sourced from 12-week-old female piglets raised on farms free from any relevant pig pathogens. Concentrations of WT, Δ *pflB,* and CΔ *pflB* during the logarithmic growth phase were adjusted to 4 × 10^4^ CFU/mL. Mix 50 μL of *S. suis* suspension with 450 μL of healthy pig whole blood and incubate at 37°C for 1, 2, and 3 h, respectively, followed by dilution and plating. The growth factors of bacteria for each hour were calculated. Growth factors were defined as the ratio of the number of viable bacteria after incubation with healthy pig blood without inactivated complement to the number of viable bacteria before incubation [26,27].

### Galleria mellonella larvae challenge assay

*G. mellonella* were randomly divided into four groups of 10 larvae each. Overnight cultures of WT, Δ *pflB, and C*Δ *pflB* were diluted to 1 × 10^7^ CFU/mL. PBS (10 μL) was injected into the larvae by using 10 μL microsyringe (Hamilton, Reno, NV, USA) as a blank group. The remaining three groups were the experimental groups and were injected with 10 μL of WT, Δ *pflB*, and CΔ *pflB* dilutions, respectively. Larval survival was subsequently recorded at 6-h intervals for the four groups. Finally, the survival curve was obtained.

### In vivo challenge experiments in mice

Mice were randomly divided into six groups, 30 in each. The groups were based on different challenge routes: intravenous infection and intranasal infection [17,28]. Adjust the concentrations of WT, Δ *pflB*, and CΔ *pflB* to reach 5 × 10^5^ CFU/mL and 5 × 10^6^ CFU/mL, respectively, for intravenous and intranasal infections. For intravenous infection, 100 μL-sample of PBS containing approximately 5 × 10^5^ CFU bacteria was given through the tail vein by 1 mL syringe. For intranasal infection, 10 μL-sample of PBS containing approximately 5 × 10^6^ CFU bacteria was given through nostrils infection by using 10 μL microsyringe (Hamilton, Reno, NV, USA). The mental status of mice in all groups was monitored at 12-h intervals for 72 h, and the average clinical score for each mouse was calculated based on defined scoring criteria. For the intravenous infection group, five mice were randomly selected for euthanasia at 12, 36, 48, and 72 h after infection to determine their blood bacterial load. For the intranasal infection group, five mice were randomly selected for euthanasia at 12, 24, 48, and 60 h after infection to determine the bacterial loads in the throat, trachea, lungs, and blood. The clinical scoring criteria were 0 for lively demeanor, active movement, climbing, and good appetite. 1 for reduced activity, less climbing, less diet intake, and mouth breathing. 2 for slightly arched back, closed eyes, limited activity and eating, drowsiness, and fast mouth breathing. 3 for messy hair with needle-like clustering on body surface and extreme drowsiness. 4 for breathing difficulty with shaking and curled-up posture. 5 for death.

### Statistical analysis

Experimental data are presented as mean ± standard deviation (SD). All experiments were repeated at least three times, and one-way analysis of variance (ANOVA) and two-way analysis of variance were performed using GraphPad Prism 9 (GraphPad Software Inc). Statistical analysis revealed significant difference (ns: no significance; *p < 0.05; **p < 0.01; ***P  < 0.001).

## Results

### PFL supported the survival of *S. suis* under glucose-limited and hypoxia conditions

*S. suis* is a facultative anaerobic pathogen, and its growth is dependent on the availability of carbon sources within its environment (Figure 1a). In this part, we investigated the growth of *S. suis* under different ventilation conditions in both glucose-sufficient and glucose-limited conditions (with galactose as the carbon source). *S. suis* WT grows better aerobically than anaerobically regardless of the monosaccharide type in the medium (Figure 1b). Also, with both aerobic and anaerobic conditions, *S. suis* WT grows better with glucose than galactose as the carbon source. Intuitively, the growth performance order of *S. suis* WT under different culture conditions from high to low is as follows: aerobic condition with glucose, anaerobic condition with glucose, aerobic condition with galactose, and anaerobic condition with galactose (Table 1b). Glucose is the preferred carbon source for bacteria, and in the presence of glucose, bacteria operate with homolactic fermentation. However, when faced with limited availability of glucose, bacteria resort to mixed acid fermentation. To evaluate the fermentation mode of *S. suis* under different culture conditions, the transcriptional levels of different node enzymes involved in pyruvate metabolism were evaluated. The expression of homolactic fermentation enzyme lactate dehydrogenase *ldh* gene was significantly higher with glucose as the carbon source than with galactose, regardless of whether it was aerobic or anaerobic. As for mixed acid fermentation, there was no difference in the expression of the pyruvate dehydrogenase *pdh* gene under the four culture conditions, while the expression of the pyruvate formate lyase *pflB* under the condition of using galactose as the carbon source was significantly higher than when glucose was used, irrespective of whether the conditions were aerobic or anaerobic (Figure 1c). It can thus be observed that PFL plays a crucial role in supporting *S. suis* survival under glucose-limited and hypoxic conditions.

### Effects of PFL deletion on metabolism of *S. suis*

**Figure 2.**
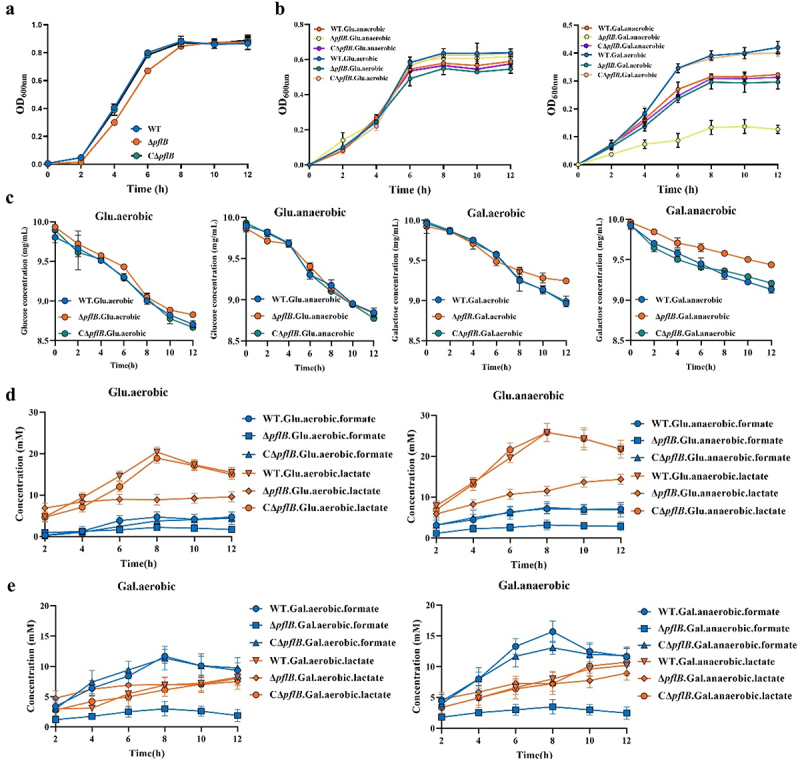
The impact of PflB knockout on the metabolism of *S. suis*. (a) Growth curves of WT, Δ *pflB*, and CΔ *pflB* under TSB aerobic culture conditions. (b) Growth performance of WT, Δ *pflB*, and CΔ *pflB* in glucose/galactose monosaccharide medium under aerobic/anaerobic conditions. (c) The residual amounts of glucose/galactose under various culture conditions. (d) Metabolites of WT, Δ *pflB*, and CΔ *pflB* in glucose monosaccharide medium under aerobic/anaerobic conditions. (e) Metabolites of WT, Δ *pflB*, and CΔ *pflB* in galactose monosaccharide medium under aerobic/anaerobic conditions. (ns: no significance; *, *p* < 0.05; **, *p* < 0.01; ***, *p* < 0.001).

PFL is crucial for the survival of *S. suis* under glucose-limited and hypoxia conditions. To further explore the role of PFL in the metabolism of *S. suis*, we constructed the PflB deletion mutant Δ *pflB* and its complementation strain CΔ *pflB*. The deletion of *pflB* has no significant effect on the growth of the strain in TSB and with glucose as the carbon source (Figure 2a). However, when galactose is used as the carbon source, the growth of the mutant strain is severely inhibited, and this inhibitory effect is more pronounced under anaerobic conditions (Figure 2b). The complementation of *pflB* partially alleviated this growth defect. Then, the residual amounts of glucose or galactose in the medium of WT, Δ *pflB*, and CΔ *pflB* under four conditions were detected to evaluate the impact of PflB deletion on the carbohydrate metabolism of *S. suis*. The deletion of PflB did not impair the ability of *S. suis* to utilize glucose in both aerobic and anaerobic environments, but it does compromise its ability to utilize galactose, particularly under anaerobic conditions (Figure 2c). Finally, the fermentation products lactate and formate in the supernatants of WT, Δ *pflB*, and CΔ *pflB* under different culture conditions were quantified to evaluate homolactic fermentation and mixed acid fermentation, respectively. In the glucose monosaccharide medium, regardless of the aerobic or anaerobic state, the main product of the WT, Δ *pflB*, and CΔ *pflB* culture supernatants is lactate. The lactate production trend of Δ *pflB* is similar to that of WT and CΔ *pflB* despite compromised homolactic fermentation in Δ *pflB* when glucose is the carbon source (Figure 2d). This shows that with glucose as the carbon source, *S. suis* mainly has homolactic fermentation, and the deletion of the *pflB* damages it to some extent. Formate, a metabolite of pyruvate metabolism by PFL, the loss of PflB function will greatly reduce the production of formate in the medium. In the galactose monosaccharide medium, formate was the main metabolite of WT, which indicated that *S. suis* mainly conducted mixed acid fermentation when galactose was the carbon source (Figure 2e). The deletion of the *pflB* greatly reduces the formate content in the medium (Figure 2e). Whether the carbon source is glucose or galactose, the complementation of the *pflB* restores Δ *pflB* to a formate level similar to WT.

Taken together, our findings suggest that PFL plays a pivotal role as a key enzyme in mixed acid fermentation of *S. suis*. The inactivation of PFL disrupts the metabolic process of *S. suis*, thereby compromising its viability under conditions of glucose-limited and hypoxia.

### Effect of PflB deletion on the morphology and stress tolerance of *S. suis*

**Figure 3.**
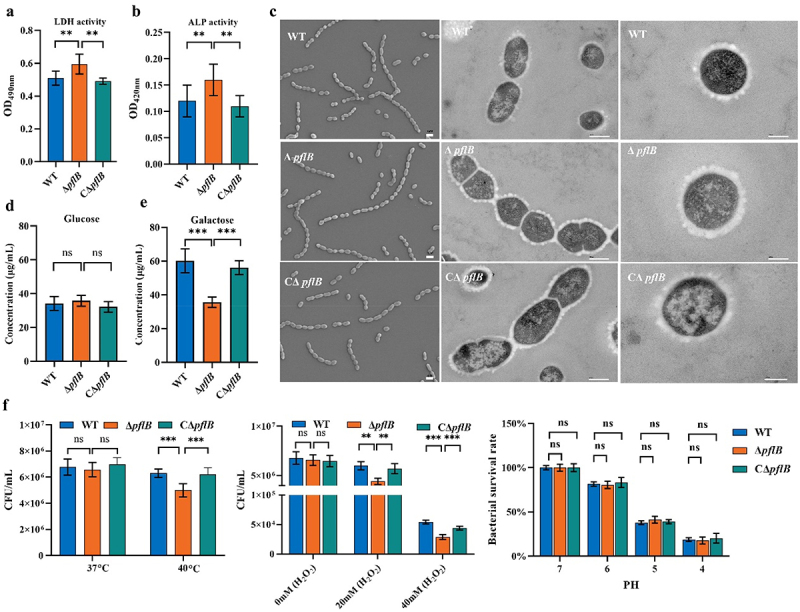
Effect of PflB deletion on the morphology and stress tolerance of *S. suis.* (a) LDH activity in culture supernatant. (b) ALP activity in culture supernatant. (c) SEM and TEM images of WT, Δ *pflB*, and CΔ *pflB*. (d) CPS content of WT, Δ *pflB*, and CΔ *pflB* under glucose monosaccharide culture. (e) CPS content of WT, Δ *pflB*, and CΔ *pflB* under galactose monosaccharide culture. (f) Growth of WT, Δ *pflB*, and CΔ *pflB*under high temperature, oxidative, and acid stress (from left to right) (ns: no significance; *, *P* < 0.05; **, *P* < 0.01; ***, *P* < 0.001).

In this part, we assessed the biological characteristics of Δ *pflB*, like its morphology, capsule, and resistance to environmental stresses. The supernatant of Δ *pflB* had higher LDH and ALP activities than the WT, but CΔ *pflB* was similar to the WT (Figure 3a, b). This means *pflB* deletion partly damages the permeability of the cell membrane and cell wall of *S. suis*. Then, SEM and TEM were performed to further investigate the changes in bacteria morphology. No significant disparity in the morphology of WT, Δ *pflB*, and *C*Δ *pflB* was observed under a magnification of 5,000× of SEM (Left panel of Figure 3c). Under TEM, WT, Δ *pflB*, and CΔ *pflB* exhibited intact and complete shapes, suggesting that although *pflB* deletion caused damage to the cell wall and membrane as detected by ALP and LDH, the damage degree was slight and not enough to affect bacteria morphology. Also, under TEM, at magnifications of 15,000× and 30,000×, the changes of the capsule of *S. suis* can be visually observed, that is, the capsule of Δ *pflB* is thicker and denser than that of WT and CΔ *pflB* (Middle and right panel of Figure 3c). Notably, the thickening of the capsule observed in Δ *pflB* was under TSB, which was complex in carbon source. The availability of sugar may exert a specific influence on capsule synthesis, particularly the synthesis of CPS, and such effects have been documented in *S. pneumoniae* [29]. To further investigate this, we examined how the loss of PflB function affects capsule synthesis under different carbon source conditions. Under glucose culture conditions, no significant differences in CPS content were found among WT, Δ *pflB*, and CΔ *pflB* (Figure 3d). In contrast, with galactose as the carbon source, the CPS content in Δ *pflB* decreased significantly compared to WT and CΔ *pflB* (Figure 3e). This shows that PflB deficiency hampers galactose utilization by *S. suis*, thereby interfering with CPS content. Finally, the ability of Δ *pflB* to resist environmental stresses, like high temperature, oxidative stress, and acid, was evaluated. The deletion of PflB significantly weakened the ability of *S. suis* to resist high-temperature stress of 40°C and oxidative stress, but did not affect the ability of bacteria to resist acidification (Figure 3f).

### Role of PFL in the virulence regulation of *S. suis*

**Figure 4.**
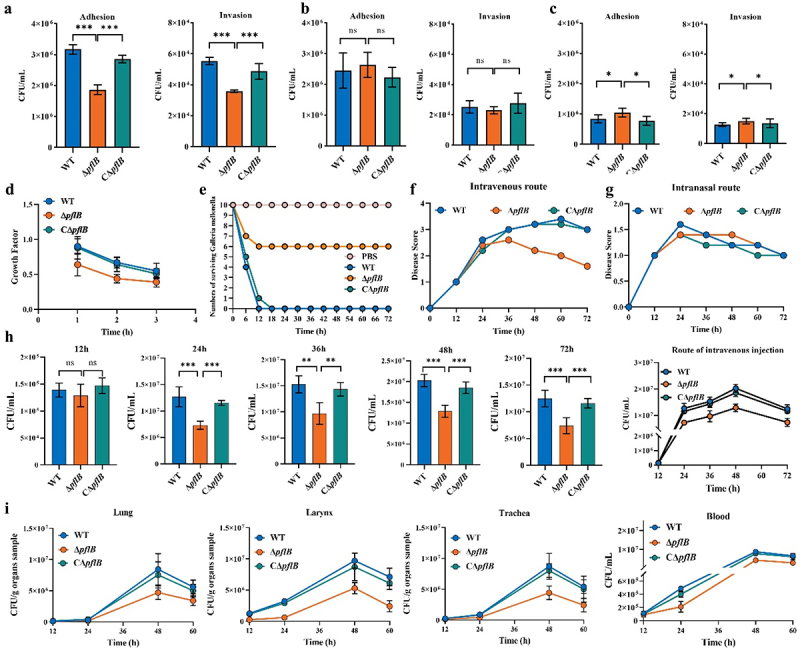
Role of PFL in the virulence regulation of *S. suis.* (a) Traditional adhesion/invasion assay. (b) Modified adhesion/invasion assay (glucose as the sole carbon source). (c) Modified adhesion/invasion assay (galactose as the sole carbon source). (d) Whole blood killing assay. (e) *G. mellonella* infection assay. (f) Daily clinical scores of mice in intravenous infection groups. (g) Daily clinical scores of mice in intranasal infection groups. (h) Evaluation of bacteremia development in intravenous infection groups (12, 24, 36, 48, and 72 h). (i) Respiratory tract colonization and the development of bacteremia in intranasal infection groups (12, 24, 48, and 60 h) (ns: no significance; *, *P* < 0.05; **, *P* < 0.01; ***, *P* < 0.001).

To investigate the regulatory function of PFL in the virulence of *S. suis*, experiments were conducted from both in vitro and in vivo. At the in vitro level, the adhesion and invasion experiments, as well as pig whole blood killing experiments, were conducted. Both traditional and modified adhesion/invasion assays were performed. In traditional adhesion/invasion assays, the adhesion and invasion ability of Δ *pflB* decreased significantly compared with WT and CΔ *pflB* (Figure 4a). Then, a modified adhesion/invasion assay was carried out to ensure that the carbon sources acquired by the bacteria were the same between the pure culture stage and the infection stage. Compared with traditional adhesion/invasion assays, the adhesion and invasion abilities of Δ *pflB* with glucose as carbon source showed no significant differences from those of WT and CΔ *pflB*. However, in the presence of galactose, Δ *pflB* showed a high adhesion and invasion ability, and there were differences between it and WT and CΔ *pflB* (Figure 4b, c). Subsequently, the ability of *S. suis* to resist pig whole blood killing was evaluated. Δ *pflB* exhibited compromised survival in pig whole blood, and this impairment was ameliorated upon restoration of the *pflB* (Figure 4d).

At the in vivo level, both *G. mellonella* and mice infection models were used. All the WT infected *G. mellonella* died within 12 h, while only 50% of the Δ *pflB* infected *G. mellonella* died within 12 h after infection. The complementation of the *pflB* partially restored the virulence of the Δ *pflB* that had been impaired; nevertheless, compared to the WT, the CΔ *pflB* still exhibited a certain degree of diminished virulence (Figure 4e). These results indicate that the loss of PflB function impairs the overall virulence of *S. suis*. Intravenous infection and intranasal infection mouse models were established to assess the impact of PflB deficiency on the ability of *S. suis* for bacteremia and chronic infection. The status of mice in the intravenous and intranasal infection groups was evaluated by daily clinical scores. In the intravenous infection groups, the disease score of mice infected with Δ *pflB* was lower than that of those infected with WT and CΔ *pflB* (Figure 4f). In the intranasal infection groups, the symptoms caused by those bacteria in mice were mild and similar (Figure 4g). In the intravenous infection groups, mice infected with WT, Δ *pflB*, and CΔ *pflB* began to develop obvious symptoms 24 h after infection, such as closed eyes and mental depression. Mice infected with WT showed symptoms of piloerection first. The symptoms of the mice infected with WT and CΔ *pflB* infection groups gradually aggravated during 24–48 h of infection, and the symptoms alleviated by 72 h post-infection. In contrast, mice infected with Δ *pflB* showed a relief at 48 h after infection (Figure 4f). Specifically, the bacterial load in venous blood was detected to assess bacteremia progression. The changing tendencies in the bacterial load in the venous blood of mice infected with WT, Δ *pflB*, and CΔ *pflB* were similar (Figure 4h). The bacterial load in the blood of infected mice peaked at 48 h after infection and then decreased. At each time point, the blood bacterial load of Δ *pflB* infected mice was lower than that of WT and CΔ *pflB* infected mice, indicating that the loss of PflB function weakened the colonization ability of *S. suis* within the blood (Figure 4h). In mice infected via the intranasal route, the chronic infection process of *S. suis* was evaluated by assessing the bacterial load in the larynx, trachea, and lung tissues at different time points after infection. We found that the colonization ability of the throat, trachea, and lung tissues was impaired in Δ *pflB* infected mice compared with that in WT infected mice. The colonization ability impairment of Δ *pflB* was largely restored in CΔ *pflB*. The bacterial load in the venous blood of mice in intravenous infection groups was also assessed. Our results showed that once the bacteria breached the mucosal barrier and entered the bloodstream, there was a diminished disparity in blood viability among WT, Δ *pflB*, and CΔ *pflB*, despite significant variations in their growth capabilities within the throat, trachea, and lungs (Figure 4i).

## Discussion

As an opportunistic respiratory pathogen, *S. suis* naturally colonizes the tonsils and nasal cavity of the upper respiratory tract without causing symptoms [1]. This asymptomatic colonization of *S. suis* can change under certain conditions, leading to invasive infection. During the transition from asymptomatic colonization to invasive infection, there is a significant alteration in the available carbon sources for bacteria, leading to a concurrent shift in bacterial metabolism, particularly within pyruvate branch metabolism [17]. However, the metabolic status of *S. suis* under different niche environments remains poorly understood. By evaluating the transcriptional levels of pyruvate node enzymes under different carbon sources, aerobic and anaerobic conditions, and the major metabolites, we conclude that PFL supports the survival of *S. suis* under glucose-limited and hypoxic conditions. The metabolic changes observed in Δ *pflB* further substantiated the pivotal role of PFL in facilitating mixed-acid fermentation. Whether at the transcriptional level or among the metabolites of pyruvate, the presence of glucose inhibited the activity of PflB in *S. suis*, regardless of whether it was in an aerobic or anaerobic environment. Conversely, under glucose-limited conditions (galactose as the carbon source), PflB shows high activity and becomes the dominant factor in pyruvate metabolism. These findings align with the metabolic regulation exhibited by PFL in *S. pneumoniae* and *S. aureus*, wherein it facilitates metabolic reprogramming under nutrient limitation and anaerobiosis [15].

Changes in metabolism also affect changes in bacterial surface structures, such as the capsule. Capsule biosynthesis is costly, depends on the metabolism and reuse of sugars by bacteria, and requires a large amount of energy [30]. Δ *pflB* cultured in TSB characterized a thickened capsule; thus, it is reasonable to suggest that PflB may be involved in the regulation of capsule synthesis in *S. suis*, but the mechanism underlying this phenomenon remains unknown. Based on the involvement of PflB in carbon metabolism in *S. suis*, we hypothesized that alterations in the capsule of Δ *pflB* might be linked to changes in its ability to metabolize different carbohydrate sources. CPS quantification in WT, Δ *pflB,* and CΔ *pflB* under different carbon sources confirmed our hypothesis. In terms of total CPS production, no significant difference was observed in CPS production between Δ *pflB* and the WT under glucose as a carbon source. However, when galactose was used as the carbon source, a significant reduction in CPS synthesis was observed in the Δ *pflB*, consistent with previous reports on *S. pneumoniae* [25].

Metabolic changes caused by PflB deficiency also affect the virulence of *S. suis*. The loss of PflB impaired the ability of *S. suis* to adhere to and invade cells in the traditional adhesion/invasion assay, which was contradicted by the modified one. The adhesion and invasion abilities of the Δ *pflB* and WT were not significantly different in the presence of glucose. However, deletion of PflB hampers *S. suis* from adhering to and invading cells under conditions where galactose is the carbon source. Similar findings have been previously reported for *S. pneumoniae* [25]. Regarding the enhanced adhesive invasion capability, our perspective is as follows. The ability of bacterial adhesion to invade cells relies on the regulation of numerous factors, with CPS serving as pivotal regulators [31,32]. The capsule was a highly hydrated anionic matrix [33]. Studies conducted on *S. pneumoniae* have demonstrated that the capsule inhibits bacterial adherence to epithelial and endothelial cells [33,34]. Similarly, in *S. suis*, the presence of the capsule interferes with the adhesion of *S. suis* to epithelial cells and macrophages [35,36]. Consequently, it is plausible that alterations at the capsule level, specifically when galactose serves as the carbon source, impede capsule synthesis while enhancing invasion. Throughout bacterial survival, the capsule exhibits dynamic changes rather than remaining static. Adherence to epithelial cells represents the initial step in the pathogenesis of infection [37]. Overcoming survival and replication challenges within the bloodstream poses a critical hurdle for *S. suis* to establish systemic infection in its host, given the presence of potent constituents of the innate immune system, such as neutrophils, the predominant immune cells residing in porcine blood, and the complement system [35,38]. In contrast to the detrimental impact of the capsule on the adhesion and invasion capabilities of *S. suis*, the presence of the capsule exerted a positive regulatory effect on the survival of *S. suis* in whole blood. CPS shield host-derived leukocytes from phagocytosis by *S. suis*, thereby enhancing bacterial evasion of immune clearance [35]. Moreover, sialic acids within *S. suis* serotype 2 CPS can disrupt the activation of the alternative complement cascade, further contributing to bacterial survival in this hostile environment [39,40]. Our findings suggest that impairment in PflB function compromises the viability of *S. suis* in porcine whole blood and warrants further investigation into the underlying mechanisms. The high glucose content in blood is well established, and no significant differences are observed in *S. suis* capsules compared to the WT when glucose exists as a monosaccharide. Based on this observation, it is reasonable to speculate that the capsule may not be the primary factor contributing to the reduced viability of Δ *pflB* in the whole blood. Therefore, further investigation is warranted to elucidate how the loss of PflB function impairs *S. suis* viability in whole blood. For *S. suis*, in vivo virulence assessment mainly relies on the mouse intraperitoneal infection model, which is also widely used by researchers. Based on the results of bacterial metabolism and in vitro infection, the critical role of different niche environments in regulating the virulence of PflB in *S. suis* was clearly recognized. By establishing intravenous and intranasal infection models, we revealed the crucial role of PflB in the progression of bacteremia and chronic infection of *S. suis*.

Overall, our study revealed the role of PFL PflB in carbohydrate metabolism in *S. suis* and indicated the role of PflB in bacteremia and persistent infection of *S. suis*, providing strong evidence for the relationship between metabolic regulation and *S. suis* virulence. In addition, our study underscores the importance of considering the context of host–pathogen interactions when assessing bacterial virulence.

## Supplementary Material

Supplementary Material 1.pdf

## Data Availability

The data that support the findings of this study are openly available in Figshare at https://doi.org/10.6084/m9.figshare.25997662
